# Effectiveness of group arts therapies (art therapy, dance movement therapy and music therapy) compared to group counselling for diagnostically heterogeneous psychiatric community patients: study protocol for a randomised controlled trial in mental health services (the ERA study)

**DOI:** 10.1186/s13063-023-07232-0

**Published:** 2023-08-26

**Authors:** Catherine E. Carr, Emma Medlicott, Richard Hooper, Yan Feng, Borislava Mihaylova, Stefan Priebe

**Affiliations:** 1 Unit for Social and Community Psychiatry, Centre for Psychiatry and Mental Health, Wolfson Institute of Population Health, Queen Mary University of London, London, UK; 2 East London NHS Foundation Trust, London, UK; 3 Barts and the London Pragmatic Clinical Trials Unit, Centre for Evaluation and Methods, Wolfson Institute of Population Health, Queen Mary University of London, London, UK

**Keywords:** Arts therapies, Art therapy, Dance movement therapy, Music therapy, Counselling, Group, Randomised controlled trial, Mental health, Economic evaluation, Process evaluation

## Abstract

**Background:**

Arts therapies are widely but inconsistently provided in community mental health. Whilst they are appealing to patients, evidence for their effectiveness is mixed. Trials to date have been limited to one art-form or diagnosis. Patients may hold strong preferences for or against an art-form whilst group therapies rely on heterogeneity to provide a range of learning experiences. This study will test whether manualised group arts therapies (art therapy, dance movement therapy and music therapy) are effective in reducing psychological distress for diagnostically heterogeneous patients in community mental health compared to active group counselling control.

**Methods:**

A pragmatic multi-centre 2-arm randomised controlled superiority trial with health economic evaluation and nested process evaluation. Adults aged ≥ 18, living in the community with a primary diagnosis of psychosis, mood, or anxiety disorder will be invited to participate and provide written informed consent. Participants are eligible if they score ≥ 1.65 on the Global Severity Index of the Brief Symptom Inventory. Those eligible will view videos of arts therapies and be asked for their preference. Participants are randomised to either their preferred type of group arts therapy or counselling. Groups will run twice per week in a community venue for 20 weeks. Our primary outcome is symptom distress at the end of intervention. Secondary outcomes include observer-rated symptoms, social situation and quality of life. Data will be collected at baseline, post-intervention and 6 and 12 months post-intervention. Outcome assessors and trial statisticians will be blinded. Analysis will be intention-to-treat. Economic evaluation will assess the cost-effectiveness of group arts therapies. A nested process evaluation will consist of treatment fidelity analysis, exploratory analysis of group process measures and qualitative interviews with participants and therapists.

**Discussion:**

This will be the first trial to account for patient preferences and diagnostic heterogeneity in group arts therapies. As with all group therapies, there are a number of logistical challenges to which we have had to further adapt due to the COVID-19 pandemic. Overall, the study will provide evidence as to whether there is an additive benefit or not to the use of the arts in group therapy in community mental health care.

**Trial registration:**

ISRCTN, ISRCTN88805048. Registered on 12 September 2018.

## Administrative information

Note: the numbers in curly brackets in this protocol refer to SPIRIT checklist item numbers. The order of the items has been modified to group similar items (see http://www.equator-network.org/reporting-guidelines/spirit-2013-statement-defining-standard-protocol-items-for-clinical-trials/).

**Table:** 

Title {1}	Effectiveness of group arts therapies (art therapy, dance movement therapy and music therapy) compared to group counselling for diagnostically heterogeneous psychiatric community patients: Study protocol for a randomised controlled trial in mental health services (the ERA study)
Trial registration {2a and 2b}.	ISRCTN.com, ISRCTN88805048. Registered on 12 September 2018.
Protocol version {3}	30 October 2022, Version 8.0
Funding {4}	National Institute for Health and Care Research (NIHR) Health Technology Assessment Programme [grant number 17/29/01].
Author details {5a}	Catherine Carr ^1, 2^ Emma Medlicott^2^ Richard Hooper^3^ Yan Feng ^3^ Borislava Mihaylova ^3^ Stefan Priebe^1,2^ 1. Unit for Social and Community Psychiatry, Centre for Psychiatry and Mental Health, Wolfson Institute of Population Health, Queen Mary University of London, London, UK. 2. East London NHS Foundation Trust, London, UK. 3. Barts and the London Pragmatic Clinical Trials Unit, Centre for Evaluation and Methods, Wolfson Institute of Population Health, Queen Mary University of London, London, UK.
Name and contact information for the trial sponsor {5b}	East London NHS Foundation Trust Contact person of the above sponsor organisation is: Angela Williams 1^st^ Floor, Bloomsbury Building St Pancras Hospital 4 St Pancras Way, London NW1 0PE Phone: 020 3317 3045 Email: sponsor.noclor@nhs.net
Role of sponsor {5c}	East London NHS Foundation Trust is the study sponsor. Noclor Research Support Service is acting on behalf of East London NHS Foundation Trust to assume overall responsibility for the initiation and management of the study. This study is funded by the National Institute for Health and Care Research (NIHR) Health Technology Assessment programme (project reference 17/29/01). The views expressed are those of the authors and not necessarily those of the NIHR or the Department of Health and Social Care. The study sponsor and funder played no part in the study design, collection, management, analysis and interpretation of the data; writing of the report; and the decision to submit the report for publication.

## Introduction

### Background and rationale {6a}

Arts therapies are widely but inconsistently provided across National Health Service (NHS) mental health trusts. Art, music and dance movement therapies can enable patients with mental illness to identify difficulties and strengths through use of the art-form in varying group interactions, facilitate emotional expression in creative activities, allow experiences and learning in non-verbal and verbal communication, strengthen self-esteem in art production (e.g. painting, song, dance) and help exploration of new emotional and cognitive approaches with the support of the group and the therapist [1]. A core principle in group therapies is the composition of group members to ensure heterogeneity of problems and experiences [2, 3]. A mix of different perspectives and experiences therefore allows greater opportunities for new behaviours and learning between group members.

To date, effectiveness trials of group arts therapies have mostly focused upon patients with a single psychiatric disorder with mixed results [4–12]. The focus on a single disorder limits the extent findings that can be generalised to how arts therapies are routinely provided. For example, the MATISSE [9] and NESS [10] trials, whilst pragmatic in nature, both focused on narrowly defined populations (patients only with schizophrenia) that are rarely encountered in usual clinical practice. In contrast, a study of individual music therapy for patients with low therapy motivation [13] is one of the few trials to have included a diagnostically heterogeneous population but did not examine this within a group context. One further study for psychiatric outpatients with severe mental illness [14] utilised group music therapy only with a songwriting focus and found significant effects on quality of life.

This study is based on the concept that therapeutically effective processes are common to all arts therapies modalities [1, 15–20]. Accordingly, NICE combines the evidence for them in one analysis [21]. However, for any arts therapy to have an effect, patients need to engage with the group and the art form used. The appeal of art forms and patient preferences vary. A poor match of preference and the offered art form is likely to lead to poorer attendance and—even in attending patients—poorer outcomes, as suggested by both MATISSE and NESS trials [9, 10]. A similar picture is seen in wider psychological treatment, preference matching is associated with lower dropout and higher therapeutic alliance [22], whereas lack of preference matching has been associated with lower perceived benefit from treatment [23–25]. Also, it does not really reflect the clinical reality as patients, if they have a choice, rarely accept a form of arts therapy that does not strongly appeal to them.

We will test whether arts therapies provided in groups are effective for diagnostically heterogeneous patients in mental health services. Patients will choose which of the three forms of arts therapy they want to participate in, thus providing modality ‘preference strata’ within which patients will be randomised to either their preferred form of arts therapy or an active group counselling control. They will be offered 40 sessions over a 5-month period. Outcomes will be assessed at baseline, at the end of treatment, and after 6- and 12-month post-intervention follow-up periods. Overall, the study will provide pragmatic evidence for the effectiveness and cost-effectiveness of group arts therapies as they are most commonly provided within NHS community mental health services.

## Objectives {7}

The primary objective is to test the effectiveness of manualised diagnostically heterogeneous group arts therapy on reducing psychological symptoms in patients receiving treatment in community mental health services as compared to an active control of group counselling (both intervention groups will be in addition to treatment as usual). Secondary objectives are to:

1. Apply stop/go criteria half-way through data collection to ensure recruitment and therapist adherence to the intervention are sufficient to continue the trial.
2. Test the effectiveness of group arts therapy on observer-rated symptoms, quality of life and objective social situation (secondary outcomes).
3. Test whether effects on primary and secondary outcomes hold true at 6- and 12-month follow-up periods post-intervention.
4. Explore the impact of adherence (completers vs. non-completers, adherence of therapists to the manual), diagnosis and type of arts therapy upon outcomes in sub-group analyses.
5. Explore processes in the above sub-groups in a nested process evaluation utilising treatment fidelity analysis, attendance data, measures of patient appraisal and experiences in the groups, and qualitative interviews exploring subjective experiences and attributions for change from the perspective of patients and therapists.
6. Assess the cost-effectiveness of group arts therapies in an economic evaluation alongside the trial.

## Trial design {8}

A pragmatic, two-arm randomised controlled superiority trial comparing group arts therapy (art, dance movement, music) to an active group counselling control, with economic evaluation and nested process evaluation. Participants will be randomised in batches, each batch sufficient in size to populate two therapy groups within a given preference stratum at a site. Randomisation will be 1:1 unless a batch has fewer than 15 participants, in which case a 2:1 allocation ratio of intervention to control will be applied.

## Methods

### Study setting {9}

The study is a multicentre trial, with five sites across the National Health Service (NHS) in the United Kingdom (UK). Recruitment and data collection will take place within secondary mental health care NHS trust community services including community mental health teams (CMHTs), recovery teams, assertive outreach teams (AOT), early intervention services (EIS) and extended primary care liaison teams within secondary care. Two study sites are inner city, two cover city suburbs, and one site covers a more rural town.

### Eligibility criteria {10}

**Patient participants**

*Inclusion criteria*

- Outpatient in secondary mental health care
- Motivation to attend group arts therapy for 5 months and expression of preference for one of three forms
- 18 years of age or above
- Primary diagnosis of ICD-10 F2 (schizophrenia and related psychotic disorders); F3 (mood disorders); F4 (anxiety and other non-psychotic disorders)
- Duration of current mental disorder of 6 months or longer
- At least moderate symptom level on the Brief Symptom Inventory (BSI) (score of 1.65 or above on Global Severity Index (GSI)) [26]
- Capacity to provide informed consent

*Exclusion criteria*

- Primary diagnosis of organic mental disorder (ICD-10 F0), substance misuse (F1), or personality disorder (F6)
- Duration of current mental disorder < 6 months (i.e. patients with short-term crises)
- Physical condition that prevents attendance of groups
- Insufficient command of English for communication with other group members and therapists.

**Therapist qualitative interviews**

*Inclusion criteria*

- Therapist providing arts therapy or group counselling as part of the trial
- Capacity to provide informed consent

### Who will take informed consent? {26a}

Recruitment will take place across secondary care community mental health teams and service-user and carer groups within participating NHS Trusts. Clinical Studies Officers (CSO) will assist the Trial Manager and Research Assistants with identification, approaching, informing and recruiting patients into the study. To aid retention, the research team will provide regular updates to the participant in the time between their initial recruitment to the study and the start of the groups.

Those with delegated roles for informed consent are the Trial Manager, Research Assistants and CSOs. The Chief Investigator retains overall responsibility for the informed consent of participants and will ensure that all those with delegated responsibility are authorised, trained and competent to participate according to the protocol, principles of Good Clinical Practice (GCP) and Declaration of Helsinki [27].

**Participant identification**

Clinicians and CSOs will screen the caseload of clinical teams to identify potentially eligible participants via medical records and review against the eligibility criteria. Those deemed potentially eligible will be contacted by a member of the clinical team either (a) during attendance of routine appointments where they are provided with a handout for potential participants or (b) via telephone or letter contact. The clinician will provide them with information about the study and obtain assent to be contacted by a member of the research team. Those who assent will have their information passed on to researchers/CSOs. The researcher will then make contact using the patient’s preferred contact method.

The researcher will go through the information sheet and answer any questions or concerns raised. If the patient is interested in participating, the researcher will then confirm contact details and arrange to meet to obtain informed consent, complete eligibility screening and, if eligible, baseline measures.

Patients linked to service-user and carer involvement groups across NHS Trusts will also be made aware of the study through the attendance of a researcher at their local meetings, who will provide information as outlined above. Any patients interested in the study will be provided with an information sheet or handout for potential participants which contains contact details of the local study team. If interested patients are ineligible, the researcher will thank them for their interest and advise them to contact their healthcare professional for further signposting to arts therapies and group therapies within the NHS Trust.

**Meeting to take informed consent, eligibility check and complete baseline measures**

All patients who express interest will be invited by phone or letter to attend a face-to-face or online meeting with a researcher. Researchers will go through the information sheet and take time to answer further questions or concerns.

If face to face, informed consent will be taken after a minimum of 24 h after first discussing the study with the researcher. All participants will be asked to provide written informed consent by initialling, signing and dating an informed consent form prior to any data collection commencing. This includes consent to video-recording of therapy sessions. Participants will be free to withdraw at any time without giving reasons and without prejudicing any further treatment.

For remote meetings, participants will be emailed or posted the Patient Information Sheet and blank consent form and then contacted by the researcher. The researcher will confirm if the participant has received the documents and will fully inform them about the study. If the participant agrees to enrol, the researcher will take verbal consent by filling in a copy of the remote consent form, which includes a note that consent is taken verbally and that the participant can contact the research team if they wish to withdraw at any point. The researcher will clearly sign, date and note that consent was taken verbally. The researcher will post a copy of the signed consent form to the participant and file a copy. Where participants have access, the consent will take place over a video-based teleconference system. The participant will initial and sign the form electronically. The signed form will be emailed to the researcher and the researcher will email a copy of the completed consent to the participant.

Once informed consent has been given, patients will be invited to complete the Brief Symptom Inventory (GSI score > 1.65) to screen for current symptom severity. If the patient scores greater than 1.65, video clips of arts therapies will be shown and the participant will be asked to state their preference for one single modality. Further baseline measures are then completed. Should the patient not meet the inclusion criteria, this will be explained and they will be thanked for their time and interest, with recommendations to speak with their healthcare professional should they wish to access arts therapies or group therapist within their service.

### Payment

Patients who attend the informed consent and eligibility check will be offered £10 to acknowledge travel and time taken in addition to their normal care visits. Those who consent and attend baseline and follow-up assessments (post-intervention, qualitative interview, 6 months and 12 months) will be offered £20 to acknowledge the time taken to travel and complete each of the assessments.

### Additional consent provisions for collection and use of participant data and biological specimens {26b}

Participants are asked to choose whether they wish to be invited to an optional qualitative interview, to have their data used in future ancillary studies and to give their consent for contact for future studies as part of the informed consent process. Participants will be assured that they do not have to consent to any of these and participation in the current trial and their existing care will not be affected. A separate section is marked optional on the consent form to make this clear. No biological specimens are collected. 

### Interventions

**Explanation for the choice of comparators {6b}**

We hypothesise that the main effect of specific arts forms in the arts therapies groups is to increase the appeal of attending such a group and to facilitate engagement through creative means when a group is offered. We are using person-centred group counselling as an active control for those effects that are outside of our model, i.e. the provision of groups, the attention from professional staff and fellow group members, and the possibility of group interactions and exchange of experiences without using arts forms.

**Intervention description {11a}**

**Group arts therapy**

Group arts therapy for the purposes of this trial comprises group art therapy, dance movement therapy and music therapy. All modalities are commonly provided within NHS mental health care. Through consultation with service users and arts therapists, we described the practice of the intervention in a manual and developed a 3-day training for therapists joining the trial [28]. All groups will consist of an opening check-in and warm-up before proceeding to a more focused use of the arts materials, with spaces to reflect upon the experience. Space will be given at the end of the session to reflect on group themes. Discussions will end with a closing activity.

**Regulation of arts therapies**

The titles ‘Art therapist’, ‘Art Psychotherapist’ and ‘Music Therapist’ are protected in the UK with requirements that an approved post-graduate course is undertaken and that the person is registered with the Health and Care Professions Council (HCPC). Art and music therapists must meet the ongoing continuing professional development and regulatory requirements and are audited on a bi-annual basis. The art and music therapists in this study will be registered with the HCPC and adhere to their requirements at all times.

Dance movement therapists do not yet have statutory regulation. The professional Association for Dance Movement Psychotherapy (ADMP) is an organisational member of the Humanistic Integrative Psychotherapy College (HIPC) which is compliant with the United Kingdom Council for Psychotherapy (UKCP) standards and regulations for practice. UKCP as an umbrella organisation is compliant with the Professional Standards Authority (PSA). ADMP also maintains HCPC standards with the aim to finalise the registration process with this regulatory body.

**Person-centred group counselling**

Group counselling will be provided based on person-centred principles. Through consultation with patients and counsellors, we amended the arts therapies manual to briefly describe group counselling for the purposes of this study and developed a 2-day training for therapists joining the trial [27]. All groups will consist of an opening welcome and introductions before proceeding to more informal discussions on topics raised by group members. Space will be given at the end of the session to have a closing activity to summarise discussions and say goodbye. The venue for these groups will be similar to those used in the arts therapy groups and, where possible, will make use of the same space. Group counselling sessions will specifically not make use of arts activities during the sessions.

**Regulation of group counsellors**

Group counsellors do not yet have statutory regulation. The British Association for Counselling and Psychotherapy (BACP) and UKCP both maintain standards and regulations for practice and are compliant with the PSA. Group counsellors will be recruited for the purposes of this study and will be required to have post-graduate person-centred counselling qualification, registration with BACP or UKCP alongside experience of providing group counselling in NHS secondary mental health services.

**Therapy schedule**

Both group arts therapy and counselling will be provided twice per week for 20 weeks with a maximum of 40 available sessions. Participants will be invited to meet individually with the therapists in the group space in the 2 weeks prior to the group commencing to discuss any concerns regarding the groups or answer any questions they may have. Each session will last between 60–90 min, comprising a 60-min focused treatment group with up to 15 min either side to afford social activity between group members arriving and leaving. Participants will be actively encouraged to attend, but are free to choose not to. Should a participant miss a scheduled session, the therapists will contact the participant to ascertain the reason for missing the session, check on their wellbeing and refer onto the clinician responsible if concerns are raised. After the final therapy group, participants will be offered an individual end of therapy meeting with the therapists to enable signposting and referral onto further services if needed.

If too few patients choose one form of arts therapy to form a group at one of the sites, or if too many study participants show poor attendance or drop out completely during treatment, we will aim to refer additional patients from outside the trial so that a critical mass of 4 or more patients is maintained in each group. To ensure a critical mass of group membership for the trial duration, if group attendance falls below 4 for 2 sessions, therapists will inform the Trial Manager and work with local services to refer additional non-trial participants into the group. New referrals must meet the inclusion criteria as defined for this study; this will be confirmed through the completion of screening measures. Additional referrals into the group will be accepted until week 10 of the intervention, after which point, the group will be closed to new referrals. Patients joining the group for this purpose will be provided with an information sheet about the study and written informed consent will be obtained for the purposes of audio-visual recording.

**Criteria for discontinuing or modifying allocated interventions {11b}**

Participants will be withdrawn from the intervention if the participant becomes too unwell to continue group participation either through:

- Loss of capacity to consent to group attendance.
- Level of risk assessed by the clinical team to require hospitalisation.
- Arts therapists/group counsellors and clinical team assess the current mental state, behaviour or risk to self or others that requires discontinuation of group attendance

It is always within the remit of the physician responsible for a patient to withdraw a patient from a trial for appropriate medical reasons, be they individual adverse events or new information gained about a treatment. If a participant chooses to withdraw from the intervention, they will be asked if they wish to continue to participate in the study and provide follow-up assessments or to withdraw from the study as a whole. Reasons for and date of withdrawal from the intervention or study as a whole will be recorded in the case report form. If a participant withdraws, we will not replace them within this study, but based on group timing and regular attendance, may open the group space to a non-trial participant to ensure a critical mass of group members is maintained. Should a participant recover or wish to recommence group therapy whilst the group is ongoing, the therapist will liaise with the clinician responsible for the participant’s care to ensure the person is ready and able to recommence. The date the intervention is recommenced will be recorded in the case report form.

**Strategies to improve adherence to interventions and assessment of compliance {11c}**

Based on a meta-analysis of music therapy for mental illness [29] and Cochrane review of dance movement therapy for depression [5], we would expect a medium effect after 20 sessions and a large effect after 40 sessions. We will take attendance of 20 sessions (50% of all available sessions) as a minimum for compliance. Compliance with the intervention will be assessed by the therapists providing the group therapy recording attendance and reasons for non-attendance on the attendance log. Late arrivals and early departures will be noted with the time and any reason for this. Should a participant miss a session, the therapists will contact them (as outlined above). Persistent noncompliance will not lead to withdrawal from the study unless requested by the participant. Measures to improve compliance will consist of:

- Meeting the therapist individually at the group location in the week prior to the intervention to set expectations and explain the intervention.
- Written reminder of the group schedule and telephone call the day prior to the group to remind the group is commencing.
- Telephone call reminders offered to participants for the duration of the study.
- Telephone call follow-up by the therapist when a session is missed.
- Buddy system of travel where participants support each other to travel to sessions together.

**Relevant concomitant care permitted or prohibited during the trial {11d}**

Participants will continue with concomitant medication and any other therapies as usual. If concerns are raised regarding the burden or interaction effects of attending both trial intervention and a talking therapy, the participant will be advised to speak with their clinician to ascertain whether or not to continue in the study.

Provisions for post-trial care {30}

This trial will adhere to the Declaration of Helsinki 2013 with respect to provisions for post-trial access for all participants who still need an intervention identified as beneficial in this trial. Clinical need and signposting to existing services have been incorporated into the treatment manuals for this trial. Participants will be offered the opportunity to meet with therapists once after the group has finished to discuss the remaining needs and services which they may wish to be referred into. Referrals will be made in conjunction with the participants’ existing care team. Any participants wishing to continue group arts therapy will be referred to existing groups within NHS community teams or offered within local charitable organisations (e.g. MIND). The study team is well integrated within the local NHS Trust care systems and will liaise on an ongoing basis with participants’ care teams in relation to any important information required by or important to care staff.

**Outcomes {12}**

**Primary and secondary outcomes {12a}**

Quantitative outcomes will be collected at baseline, within 4 weeks of the end of the intervention (post-intervention) and 6 and 12 months post-intervention ending. The primary outcome will be psychological distress at end of treatment (20 weeks), measured using the Brief Symptom Inventory global severity index (GSI) [26]. Our secondary outcomes are as follows: Brief Symptom Inventory (BSI) subscales: Somatisation, Obsessive compulsive, Interpersonal sensitivity, Depression, Anxiety, Hostility, Phobic Anxiety, Paranoid Ideation, Psychoticism and Positive Symptom Distress Index; observer-rated psychiatric symptom severity on the Brief Psychiatric Rating Scale (BPRS) [30]; quality of Life on the Manchester Short Assessment of Quality of Life Scale (MANSA) [31]; and objective Social Situation (SIX) [32].

**Treatment fidelity {12b}**

We will take both therapist and observer ratings of adherence to the manual. Therapist self-rated adherence to the manual will be rated by therapists at the end of each session on an adherence form designed for the purposes of this study. Observer ratings of treatment fidelity will be made from videos of 10% of therapy sessions, selected at random and rated on the adherence form designed for the purposes of this study.

**Process evaluation {12c}**

A process evaluation will consider the following data and measures including, attendance of therapy sessions and reasons for nonattendance over the 20-week intervention period. Patient-reported appraisal of the sessions in weeks 2, 7, 12 and 17 of the intervention period on the Outcome Rating Scale (ORS)  [33]. Self-reported depression in weeks 2, 7, 12 and 17 of the intervention period on the Personal Health Questionnaire (PHQ-9) [34]. Group experiences in weeks 2, 7, 12 and 17 of the intervention period on the Ferrara Group Experiences Scale (FGES) [35].  Qualitative end of therapy interviews with a purposive selection of 13% of participants and therapists using the Client Change Interview [36].

**Economic evaluation**

Economic evaluation will include the following measures at baseline, post-intervention, 6 months and 12 months post-intervention: quality of life for trial participants on the EQ-5D-5L [37], Recovering Quality Of Life (ReQoL-20) [38]; use of health and social care services on the Client Services Receipt Inventory (CSRI) [39]; and costs estimation of group arts therapies (art therapy, dance movement therapy and music therapy) and group counselling using inventory forms designed by the trial Health Economist.

### Participant timeline {13}

A participant timeline summary can be found in Table 1, including the schedule for enrolment, intervention and assessments.

**Table 1:**
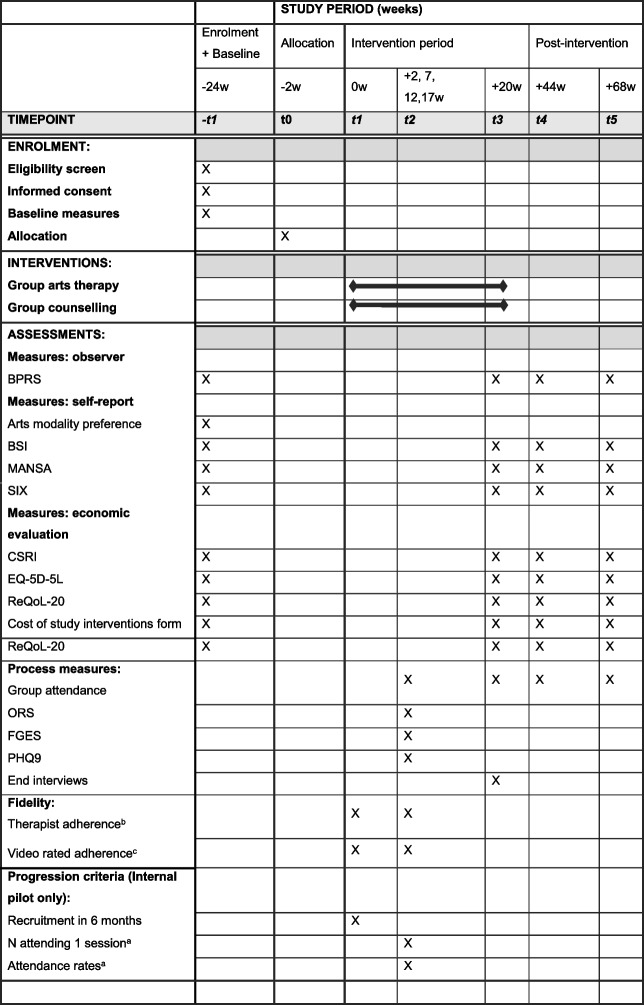
Schedule of procedures (SPIRIT diagram)

^a^Measured in the first 4 weeks of the intervention period

^b^Therapist self-rated adherence measured every session

^c^Video-rated adherence from one session in weeks 2 and 3 per therapy group will be used for the internal pilot; 10% of sessions will be randomly selected for the process evaluation

### Sample size {14}

We have designed the trial to detect a treatment effect of 0.5 standard deviations on the primary health outcome, i.e. the level of psychological distress, as measured by the Brief Symptom Inventory Global Severity Index. In a sample of 378 patients from a UK psychiatric outpatient population, the mean GSI was 1.65 with a standard deviation of 0.81 [40]. An effect of 0.5 standard deviations would therefore represent a difference of 0.4 on the GSI. We assume clustering of outcomes of patients treated in the same therapy group. In the NESS trial on group body psychotherapy [10], the ICC for therapy groups of 10 patients varied for different outcomes, but did not exceed 0.01 (which applied to the primary outcome). We assumed, conservatively, an ICC of 0.1. We assume a drop-out rate of 15% by the end of the study, so that if we allocate 10 patients on average to each therapy group we will end up with clusters with 8.5 patients on average. Assuming a coefficient of variation of cluster size of 0.5, we will need 200 patients before drop-out in 20 clusters in each arm to achieve 90% power at the 5% significance level [41]. Allowing, conservatively, for the additional loss of one full cluster in each arm, we plan to recruit a total of 2 × 210 patients.

The estimated loss takes into account drop-outs at each of the different phases of the study, i.e. between consent and beginning treatment, during treatment, during the 6-month follow-up period and during the 12-month follow-up period. Some patients will have to wait between giving consent and beginning of treatment. Within this period, they may drop out due to changing their mind, being offered alternative treatment or experiencing a reduction in symptoms. Based on previous studies [9, 10], we expect this drop-out rate to be less than 10% and we will be able to compensate for most of that loss through recruiting additional patients closely before the time of the baseline assessment.

During the treatment phase, we may have drop-outs from both groups. An intention-to-treat approach to analysis does not mean that all outcome data must have been collected, but it does mean that every effort should be made to minimise the amount of missing data. We expect a low drop-out rate from research during treatment but envisage a drop-out rate of 10% by the 6-month follow-up (in NESS [10] it was 7.3% after 6 months), and 15% by the 12-month follow-up.

### Recruitment {15}

We plan to recruit from Secondary Mental Health care services including CMHTs, Recovery teams, and Secondary Care service user and carer involvement forums within the participating Trusts. To ensure adequate participant enrolment, we have begun by engaging with Senior Clinical leadership within each NHS Trust and then wider management teams to agree on the most effective strategies from which we will recruit. We have built in flexibility for different recruitment methods (letter, in-person and clinician approaches) alongside flexibility for research meetings to take place (on clinical sites, at participants’ homes or remotely).

For the first phase of recruitment (in the first 6 months leading up to the stop–go decision point), based on a participation rate of 25%, we plan to approach 240 patients at each site, with the aim of recruiting 10 patients per month per site over 6 months (180 patients in total). In the second phase of recruitment, we plan to double the number of groups in London by including an additional site. For this, based on a participation rate of 25%, we plan to approach 240 patients at each London, with the aim of recruiting 10 patients per month over 6 months.

## Assignment of interventions: allocation

### Sequence generation {16a}

Randomisation will be on a site-by-site basis (three sites in the first phase of recruitment, and four sites in the second stage) and will be further stratified at each site by preferred modality. To minimise the time between enrolment and commencing the intervention, we will aim to conduct randomisation as soon as a batch of 20 people with the same modality preference have been enrolled at a site and have completed baseline measures. Participants in each batch of 20 will be randomised in a 1:1 ratio to two therapy groups, one receiving the preferred modality and one receiving group counselling (the control), using constrained randomisation to balance the distribution of primary diagnosis, age and gender in each therapy group. We implement constrained randomisation using the cvrand command in Stata [42].

If 15–19 participants with a preferred modality are still to be randomised at the end of the recruitment window, they will be randomised 1:1 to two therapy groups, with the possibility to add non-study participants to maintain numbers within the group. In the case where there are fewer than 15 participants with a preferred modality at the end of the recruitment window, they will be randomised (where feasible) in a 2:1 ratio to intervention and control, with the option of enlarging groups with non-study patients.

At the end of recruitment, there is a chance that the number of participants with a preferred modality is below that of the number required to feasibly run a group. Taking into account drop-out and non-attendance, we estimate that we require a minimum of 8 participants for a group to be viable. This equates to a minimum of 12 participants in a randomisation batch if randomising 2:1. In order to minimise the loss of already consented participants, we will ask participants to provide a second modality choice and also to indicate whether or not they would be willing to receive this in case numbers for their original preferred modality cannot be met.

Where the number in a preference stratum at the end of recruitment is fewer than 12, we will consider enriching this stratum with participants for whom the modality was their second choice, to give a sufficient number to be randomised as a batch. If a participant is unwilling to receive a second choice, we will withdraw them from the study and signpost them to local services.

**Concealment mechanism {16b}**

Randomisation is performed in a data safe haven by an independent statistician at the Pragmatic Clinical Trials Unit. The allocations are passed back to the unblinded trial manager via secure file transfer protocol who then informs the therapists and principal investigators at each site. Allocation status is saved in password-protected files, held in folders that are inaccessible to blinded members of the research team.

**Implementation {16c}**

Each newly recruited participant’s details will be entered by researchers into a database once they are enrolled and baseline data have been collected. When a batch of participants in a preference stratum at a site is ready for randomisation, the trial manager sends this file to an independent statistician in the Pragmatic Clinical Trials Unit who will then randomise participants as described under Sequence Generation, above.

## Assignment of interventions: blinding

### Who will be blinded {17a}

All research assistants completing follow-up assessments, one co-CI (Priebe), the trial health economist and the trial statistician will be blinded to the treatment allocation until all follow-up assessment data is collected and the statistical and health economics analysis plans are signed off.

Prior to each meeting with research assistants, participants will be reminded by an unblinded member of the research team not to disclose any details of the intervention in which they took part. In the event of unblinding, this will be recorded, specifying whether or not this occurred before or after the primary outcome measure (BSI) was completed. Should the research assistant be unblinded, future assessments will be allocated to a different blinded research assistant where possible. Given the nature of this trial, it is not possible to blind the participants, arts therapists or group counsellors to the arm of the trial they are in as there will be obvious differences due to the presence or absence of arts activity in sessions. One CI (Carr), the Trial Manager, PIs for each site and members of the treating health-care team will not be blinded. One CSO per site will be unblinded to record process measures during the intervention phase.

### Procedure for unblinding if needed {17b}

Patients, therapists providing the intervention, PIs for each site, one CI (Carr) and members of the health care team are not blinded to the intervention. Should a patient need to be withdrawn from the study due to clinical concerns, this will be logged by the therapists, and the Trial Manager informed. Follow-up assessments will continue to be conducted by a blinded researcher if the participant is happy for this to continue and continues to have capacity to consent. Study code will only be broken if there is a severe adverse event (SAE) where it is necessary for the blinded co-CI to know which intervention the service user is receiving.

- The CI documents the breaking of the code and the reasons for doing so on the CRF/data collection tool, in the site file and medical notes. It will also be documented at the end of the study in any final study report and/or statistical report.
- The CI/Investigating team will notify the Sponsor in writing as soon as possible following the code break detailing the necessity of the code break. 
- The CI will also notify the relevant authorities.

The written information will be disseminated to the Data Safety Monitoring and Ethics Committee (DMEC) for review in accordance with the DMEC Charter. The responsibility for providing this will be held by the unblinded CI and provision documented.

## Data collection and management

### Plans for assessment and collection of outcomes {18a}

All staff employed on the study receive annual mandatory training from their local NHS Trusts on information governance, safeguarding, health and safety, manual handling and infection control, and work within national and international legislation in these areas. The study team will be trained in all aspects of GCP and informed consent processes via the study sponsor (Noclor). Researchers conducting assessments will be trained in how to conduct the Brief Psychiatric Rating Scale interview, drawing upon the ‘structured interview guide’ [43] that has been adapted to align also with wider assessment questions asked. Data will be collected on paper case report forms and then entered into an electronic database (OpenClinica), which was developed by data managers and researchers with a thorough testing process.

**Validated assessment scales**

*The Brief Symptom Inventory* [26] has good internal consistency (Cronbach’s alpha: 0.84), sensitivity of 82% and specificity of 75% and provides information regarding symptom distress on a range of psychological symptoms.

*The Brief Psychiatric Rating Scale (BPRS)* [30] has an internal consistency that varies between 0.75 and 0.79 [44] and test–retest reliability of 0.78 [43]. Scores of 31 approximately correspond to Clinical Global Impression ratings of ‘mildly ill’, 41 as ‘moderately ill’ and 53 as ‘markedly ill’ [45]. As recommended by UCLA BPRS fidelity gold standard [46], consensus rating must be reached by each interviewer on a minimum of six interviews before research assistants could independently conduct BPRS interviews.

*The Manchester Short Assessment of Quality of Life (MANSA)* [31] has good internal consistency for satisfaction ratings of 0.74 and correlations of 0.83 and higher with the longer Lancashire Quality of Life Profile [31].

*The Objective Social Situation (SIX)* assessment was developed as a ranking scale to record social outcomes of work/employment, accommodation/housing and social situation (living situation and social contact in the last week). The scale score ranges from 0 to 6. The scale has good sensitivity to change and no floor or ceiling effects [32].

**Measures in economic evaluation**

*EQ-5D-5L* [37] is an internationally widely used generic Patient Reported Outcome Measure. The sum score ranges from 5 to 25.

*ReQoL-20* [38] is a new Patient Reported Outcome Measure that has been developed to assess the quality of life for people with mental health conditions. The sum score ranges from 0 to 80.

*CSRI* [39] is a widely used tool to comprehensively record the support and services received by participants in research studies.

**Process measures**

*Outcome Rating Scale (ORS)* [33] has high internal consistency (*α* = 0.93) and is moderately correlated with the longer Outcome Questionnaire 45.2 [47]. Designed for a clinically usable alternative to the OQ45.2, it provides a brief measure of overall, individual, interpersonal and social functioning.

*Ferrara Group Experiences Scale (FGES)* [35] has good internal consistency (*α* = 0.85) and measures the types of group experiences most prominent within a group session (both positive and negative).

*Personal Health Questionnaire (PHQ9)* [34] is a widely used, brief self-report measure of depression, with excellent internal consistency (0.86–0.89).

### Plans to promote participant retention and complete follow-up {18b}

To ensure an adequate follow-up rate, we will:

- Maintain regular contact with patients after giving informed consent (every 6–8 weeks).
- Ensure contact occurs in the 4 weeks prior to the groups starting, to ensure greater levels of contact between consent and the intervention starting.
- After randomisation, patients will be given the opportunity to meet the therapists and ask questions prior to the group starting.
- Patients will be reimbursed for their expenses and time for each research interview (£10 for consent appointment and screening if not eligible, i.e. BSI < 1.65, with a further £10 to complete further baseline questionnaires if eligible, £20 for each subsequent interview).
- Research assistants will arrange the date for the next interview during the previous assessment and use a telephone and written reminder 2 weeks prior to the subsequent interview date.
- As far as possible, the same research assistant will conduct all interviews with a patient so that a positive relationship can be established.
- We will accommodate patients’ preferences for meeting times and locations, including patients’ homes.

Where an assessment appointment is missed:

- The research assistant will follow-up non-attendees via telephone call to ascertain the reason for non-attendance and whether any assistance from the study team can help in this matter.
- The research assistant will check if the participant is still willing to continue with the study and record the participant’s response.

### Data management {19}

**Data collection and storage**

Data handling and record keeping are specified in the data management plan agreed with the PCTU, following Standard Operating Procedures (SOPs).

To prevent missing data, Research Assistants completing assessments will ensure completeness of recording data throughout the research assessment. Should missing data be discovered after the assessment, the research assistant follows up with a telephone call to the participant as soon as possible. Clear signifiers for reasons for missingness will be agreed and specified following the PCTU SOP for data management.

All data for participants will be collected by the Trial Manager or research assistants and entered into a paper Case Report Form (CRF) designed for the study using a pseudonymisation system set up a priori allocating each participant to an unrelated code number. Pseudonymised paper forms of the CRF data will be stored in a locked filing cabinet at each participating site, kept separate from the pseudonymisation code sheet identifying participants. Signed consent forms and demographic details will be kept and locked securely and separately from pseudonymised data with the pseudonymisation code kept at the Unit for Social and Community Psychiatry. Data will be backed up digitally through manual data input of pseudonymised CRFs and source documents on password-secured NHS computers and saved on password-protected and encrypted hard disks securely stored in a locked cabinet at the Unit.

**Data entry and quality assurance**

Data will be entered from hard pseudonymised copies and entered on-site into an online database by research assistants at each NHS Trust. The blank dataset will be prepared by QMUL PCTU’s Data Management team. The data will be viewable online by the central trial team.

The final datasets will be stored as Csv. files and utilised to conduct analyses by the Statistical Packages for Social Sciences and Stata software, as appropriate. The Trial Manager will be responsible for an on-going check of data quality. The research assistants and Trial Manager will double-check data entry from pseudonymised CRFs to digital data entered on a weekly basis. Sites will send copies of 10% randomly selected data files and completed questionnaires to the central research team who will carry out a review, whereby these data files will be entered again by the trial manager and subsequently compared with the data in the files received from the Research Assistants at the participating Trusts. In case of major differences, the proportion of the reviewed data will be increased. In case of a significant mismatch (more than 10%), the Trial Manager will discuss with the Research Assistants at participating sites in order to further investigatedata problems. Data quality assurance will be discussed in weekly teleconferences with the research assistants at participating sites.

**Qualitative interview data**

Data from qualitative interviews will be transcribed verbatim by an NHS-approved transcription service. The Trial Manager (or delegated to Research Assistant) will check to ensure the accuracy of the transcription, and removal of any potentially identifiable information prior to deleting the audio file.

Transcribed data will be stored in password-protected files on NHS computers with restricted access only to the research team.

**Data security**

Digital data is backed up securely every night and stored on NHS servers. The Unit/PCTU complies with the Data Protection Act 2018 and all staff involved in the study receive mandatory training on this on an annual basis. The Trial Manager, Chief Investigators and PCTU statistician and health economist are responsible for data analysis once data collection is complete. Any personally identifiable information (such as consent forms and audio-visual recordings) will be stored separately from the pseudonymised data in a locked filing cabinet on NHS premises to which only the study team will have access.

Audio visual files will be stored on password-protected and encrypted hard drives in a locked filing cabinet as above. We will seek permission from participants to use non-personally identifiable data (e.g. Music making, visual images of art-work, movement without full picture of face or body, re-recorded examples of group discussions with actors) from therapy sessions for the purposes of illustration of findings and presentation. This is outlined in the participant information sheet and consent form. All original audio-visual recordings of therapy sessions will be destroyed 1 year after the end of the study. All audio files of interviews will be destroyed immediately after transcription.

Direct access will be granted to authorised representatives from the Sponsor, host institution and the regulatory authorities to permit trial-related monitoring, audits and inspections in line with participant consent.

**Record retention and archiving**

Data will be retained and archived in accordance with the UK Policy Framework for Health and Social Care Research, East London NHS Foundation Trust Record Management and IM&T Information and security policies. All essential documents will be archived for 20 years after completion of trial and stored in the Trust Modern Records Centre. The Chief Investigators will be the custodian of the data. Participants’ contact details will be retained (with their permission via the consent form) if they want to be updated about the study progress. These will then be destroyed 1 year after the study end.

### Confidentiality {27}

The trial will be compliant with the requirements of the Data Protection Act, 2018, with regard to the collection, storage, processing and disclosure of personal information and will uphold the Act’s core principles, throughout the study.

**Personal information**

All data will be pseudonymised to maintain patient confidentiality. All participants will be assigned participant ID number used for all data processing purposes and the list linking these data with the participant ID number will be stored on NHS computers on a secure drive, within password-protected folders, which will only be accessible to the research team. All hard copies of data, including signed consent forms, socio-demographic details and patient receipts, will be kept and locked securely and separately from pseudonymised data and only accessible by the research team. Where participants provide their consent, participant’s names and contact details will be retained to enable the research team to re-approach them to take part in related studies and to share research findings.

**Pseudonymised data**

Pseudonymised CRF data will be stored in a locked filing cabinet at each site, kept separate from the anonymisation code sheet identifying participants. Data will be entered into a web-based database developed by the PCTU Data Management team. All data stored on the database will be pseudonymised by using the participant ID as the identifier. The database will be accessed by researchers working on the study.

**Audio visual recordings**

Audio-visual recordings of all therapy sessions (arts therapies and group counselling) and individual interviews will be taken with explicit permission (as indicated on the consent form) from participants. Recordings will be stored on password-protected folders on NHS Trust computers on a secure drive which will only be accessible by the research team. Audio recordings of interviews will be destroyed immediately after transcription and the transcriptions will not contain any identifiable information. Audio-visual recordings will be destroyed 1 year after the trial as finished, with only excerpts of sessions kept for presentation purposes. These excerpts will not contain any identifiable images or sounds of participants and will be used only with the explicit consent of participants (as outlined in the information sheet and consent form).

**Access to data**

Access to pseudonymised data will be limited to the following personnel who may all be involved in handling or analysing data during the study: the trial manager, CIs, service user lead, research assistants, clinical studies officers and PCTU staff. No identifying data will be sent to the sponsor or TSC/DMEC members. The only occasion where information on patients may need to be transmitted via NHS email or telephone would be in the case of a serious adverse event, where a therapist may need to contact the CI. In this case, Trust guidelines will apply, i.e. minimally identifying data will be used on the emails, NHS to NHS email only will be used and NHS guidelines for checking caller identities for phone calls will be followed.

### Plans for collection, laboratory evaluation and storage of biological specimens for genetic or molecular analysis in this trial/future use {33}

Not applicable as no biological specimens are being collected.

## Statistical methods

### Statistical methods for primary and secondary outcomes {20a}

A detailed statistical analysis plan will be drafted by the trial statisticians and signed off by the independent statistician on the Trial Steering Committee prior to the analysis of unblinded data.

Baseline data

We will report descriptive statistics for sociodemographic and clinical characteristics by intervention arm along with baseline scores for each assessment.

**Primary outcome analysis**

The primary analysis will be a longitudinal mixed regression analysis that includes end of treatment, 6-month post-treatment and 12-month post-treatment assessments of psychological distress, using all non-missing data on these outcomes. Performing a longitudinal analysis of all time-points using all non-missing data should allow for greater precision in the estimation of treatment effect at end of treatment than an analysis of this time-point alone. However, to allow results on the primary outcome to be reported in a timely fashion, we will also conduct and report an initial analysis of the first two time-points—baseline and end of treatment—as soon as these are available for all participants. The analysis will include random effects to allow for clustering within each therapy group and individual. The primary hypothesis of a beneficial effect of arts therapy will be investigated by testing for a fixed effect of intervention vs. control, adjusting for fixed effects of site, patients’ preference for the form of arts therapy (art, music or dance), primary diagnosis, age, sex and baseline psychological distress. The primary analysis will be by intention to treat. Secondary analyses will investigate the treatment effect in compliers.

**Secondary outcome analysis**

Secondary outcomes, which are all on continuous scales, will be analysed in the same way as the primary outcome.

**Economic evaluation**

Economic evaluation will be undertaken from the NHS and personal and social services perspective to assess the cost-effectiveness of group arts therapies compared to group counselling for psychiatric patients. The evaluation will include within-trial analyses over the intervention period and the 12-month post-intervention follow-up period.

We will estimate the cost of delivering group arts therapies (i.e. art therapy, dance movement therapy and music therapy) and control intervention (i.e. group counselling). Resource use associated with delivery of all interventions will be documented by the study team over the 5-month treatment period using inventory forms developed by the trial Health Economist. Data on patients’ use of health and social care services will be collected at baseline, end of treatment and 6 and 12 months after treatment by the trial researchers using an adapted CSRI [39]. Costs for each type of resource (or service) use will be calculated as a product of the quantity of resource (or service) use and its corresponding unit cost. Cost items will be summed together and presented at baseline and 12 months after intervention for each patient. The primary health outcome measure in the economic evaluation is patients’ self-reported outcomes on the EQ-5D-5L instrument [37]. We will convert the ED-5D-5L data to quality-adjusted life years (QALYs).

Cost-utility analysis will be conducted. We will calculate the incremental cost-effectiveness ratio (ICER) of the group arts therapies compared to group counselling and then check the point estimate against a cost-effectiveness threshold value of £20,000 to £30,000 per QALY [48]. We will also report the uncertainties around the point estimate. In addition to the primary analysis, a few sensitivity analyses will be conducted including to (1) undertake analysis using broader analytical perspective in the economic evaluation by including estimated costs from productivity lost; (2) run the primary analysis use intervention period alone or intervention period and 6-month period after intervention as the time horizon; (3) perform two scenario analyses using the ReQoL-20 [38] and BSI [26] as the alternative health outcome measures; (4) consider uncertainty in the intervention costs; (5) explore the pattern of missing values in costs and outcome data and then identify an appropriate method to manage missing values; and (6) estimate ICER for each form of art therapy in order to assess its cost-effectiveness compared to group counselling.

### Interim analyses {21b}

**Stop/go criteria**

As an interim assessment of feasibility, we will examine recruitment rates in the first 6 months, alongside group attendance rates and observer-rated therapist adherence to the manual, using pre-defined stop–go criteria (Table 2). We will aim to recruit 180 participants (60 at each initial site). In week 4 of the intervention phase, the stop–go criteria will be discussed by the Trial Management Group (TMG), Trial Steering Committee (TSC) and funder to decide whether or not to continue the trial. Recruitment will continue throughout this time period, with the aim of commencing the next round of groups as soon as possible after receiving this decision.

**Table 2:** Stop–Go criteria after 6 months of recruitment

**Criterion**	**Green** Progress to end of trial as planned	**Amber** Discussion with TSC and funder about progression	**Red** Trial is stopped
Recruitment within 6 months	≥ 90% of recruitment target at each site	66–89% of recruitment target at each site	< 66% of recruitment target at each site
Attendance of at least one group session in the first 4 weeks of intervention	≥ 90% of sample attend at least 1 session	66–89% of sample attend at least 1 session	< 66 of sample attend at least 1 session
Attendance rates in the first 4 weeks of the intervention	≥ 90% attendance rate	66–89% attendance rate	< 66% attendance rate
Therapist adherence to manual using observer-rated videos from sessions taken from weeks 2 and 3 of the intervention	≥ 90% agreement with manual criteria	66–89% agreement with manual criteria	< 66% agreement with manual criteria

No interim analyses of primary or secondary outcomes are planned. The DMEC will review data on outcomes and adverse events as they accrue, summarised by trial arm, arts therapy modality, study site and diagnosis. Should significant safety concerns arise during the intervention phase, the CI in liaison with the sponsor, TSC and DMEC has ultimate authority to halt the study or withdraw individual participants should concerns arise during the study.

### Methods for additional analyses (e.g. subgroup analyses) {20b}

**Subgroup analyses**

We will repeat analyses within diagnostic subgroups and investigate whether different forms of arts therapy have different effects by including an interaction between modality preference stratum and intervention vs. control in our mixed regression model.

Qualitative assessments and nested process evaluation

We plan to conduct a process evaluation in line with recommendations by the Medical Research Council [49]. The logic model of our intervention (described in our manual development paper [28]) provides a theory of the intervention describing assumptions and contextual factors that might shape implementation and outcomes, hypothesised processes and mechanisms of impact and our intended outcomes. The aim is to better understand the processes of group arts therapy in comparison to group counselling controls in practice and possible implications. In particular:

1. To understand exactly how the intervention was delivered in practice (treatment fidelity analysis).
2. Describe processes of attendance and hypothesised process factors of self-reported depression, group experiences and session appraisal over the course of the trial.
3. Understand subjective experiences and attributions for change of the intervention from the perspective of patients and arts therapists.
4. Compare reported quantitative and qualitative processes against the proposed logic model and to revise accordingly.

**Method**

The process evaluation will employ an embedded mixed methods design and will consist of data collection of video data of the intervention itself (through treatment fidelity analysis), client self-reported measures (weeks 2, 7, 12, 17 PHQ9, FE-GES, ORS) and qualitative end interviews scheduled within 6 months of the end of the intervention with participants and arts therapists. The interview is optional for participants but we aim to conduct a minimum of 45 interviews.

Quantitative analysis will provide a descriptive analysis of the course of process measures. We will descriptively explore whether there are any differences between compliant and non-compliant attenders, responders and non-responders and whether any socio-demographic and clinical characteristics are associated with outcomes.

Qualitative evaluation will comprise of one-off end-of-study interviews with participants from all 3 arts therapies modalities and control groups within 6 months of the intervention ending (weeks 20–48).

Participants will be purposively sampled so that we have a range of characteristics based on the following: treatment completers (attended > 75% of sessions) and partial-attendance (attended 35–75% of sessions), representation of each of the diagnostic groups (ICD10 F2, F3, F4), representation of a range of ages, each mode of therapy and sites. We aim to conduct interviews with up to 3 patients per arts therapies group and up to 10 control group participants, with an anticipated sample size of around 55 participants.

Interviews will last up to 1 h and will be conducted by an unblinded member of the research team. We will use an amended version of the Client Change Interview [36], which explores experiences of therapy, changes over a given time period and attributions for this. The amended version used in existing music therapy studies also explores participants’ experiences of the research process and involvement in the study as a whole. This information will provide understanding not only of the interventions but of how best to involve this patient group in trials in the future. Following advice from our LEAP, we will also interview the arts therapists providing the intervention as a means of triangulating patients’ experiences of change and relating identified changes to observations within the sessions. These interviews will also last for 1 h and will happen within 6 months of the therapy group ending.

Given the relatively large qualitative sample size due to nesting of participants within groups and diagnoses, we will initially analyse the material using the framework approach outlined by Ritchie et al. [50] whereby after familiarisation with transcripts, a thematic framework is constructed—informed by the theoretical model for this study and interview content—and then use charting within thematic matrices to examine similarities and differences on core characteristics outlined above across and within themes. We will relate this to the hypothesised theory of the intervention [28] and highlight where our data supports of conflicts with this hypothesis.

### Methods in analysis to handle protocol non-adherence and any statistical methods to handle missing data {20c}

A longitudinal regression analysis of all non-missing data will give a valid estimate of treatment effect under an assumption that outcomes are ‘missing at random’ (that is, missingness is influenced only by variables that are included in the analysis). If the primary outcome is missing for more than 5% of participants, we will conduct further analyses to investigate the sensitivity of our conclusions to departures from the missing at random assumption.

### Plans to give access to the full protocol, participant-level data and statistical code {31c}

The full protocol is available to access from the authors. Once the main findings are published, the full anonymised participant-level dataset will be made available on request.

## Oversight and monitoring

### Composition of the coordinating centre and trial steering committee {5d}

The trial has four management committees: the Trial Management Group (TMG), the independent Trial Steering Committee (TSC), the Data Monitoring and Ethics Committee (DMEC) and the Lived Experience Advisory Panel (LEAP). The TMG includes the CI, co-CI, a range of clinical co-applicants, the main researchers and patient representatives from the LEAP. The TMG will meet regularly (at least every 2–3 months) to ensure all practical details of the trial are progressing and working well, and to ensure everyone within the trial understands them. More regular and individual meetings between the PIs, site leads and different parts of the research team will be arranged as appropriate. The TSC includes an independent chair, clinician, statistician and patient representative. The TSC will meet jointly with the DMEC at the beginning of the study and then immediately following the delivery of expected major milestones. Further meetings will be arranged as and when required. The LEAP will consist up to eight patient and carer members with experience in secondary mental health services or caring for someone accessing such services. They will meet at least twice per year to advise on study materials, progress and findings including website content and development, recruitment strategy, patient-facing information, issues during the running of the trial and contribution towards analysis and interpretations of findings, including suggestions as to how to present these to wider members of the public and develop lay summaries.

### Composition of the data monitoring committee, its role and reporting structure {21a}

The DMEC will include committee members who are completely uninvolved in the running of the trial, independent from the Sponsor, and who cannot be unfairly influenced (either directly or indirectly) by people or institutions involved in the trial. The committee will include one trial methodologist, a statistician and a patient representative. They will meet immediately prior to TSC meetings (and more regularly if required).

### Adverse event reporting and harms {22}

**Adverse events (AE)**

Adverse events will be recorded in the main trial database and the participant’s clinical record, with the participant followed up by the research team.

**Serious adverse event (SAE)**

SAEs will be recorded as above, with the additional completion of serious incident form of the NHS Trust, and notification of the Sponsor within 24 h of research staff becoming aware of the event. Those that are assessed as ‘related’ and ‘unexpected’ will be reported also to the Research Ethics Committee (REC) within required expedited reporting timescales. All deaths will be reported to the Sponsor irrespective of whether the death is related to disease progression, the intervention, or an unrelated event within 24 h of the death becoming known by the research team.

**Urgent safety measures**

If any urgent safety measures are taken, the CI/Sponsor will give written notice to the REC within 3 days of the measures taken and the circumstances giving rise to those measures.

**Overview of safety reporting responsibilities**

The PIs will ensure that local safety monitoring and reporting are conducted according to the Sponsor’s requirements. The CI holds clinical oversight of the safety of patients participating in the trial. The Trial Manager is responsible for reporting all AEs and SAEs to the DMEC on an at least 3 monthly basis. The DMEC and TSC are responsible for periodically reviewing safety data and liaising regarding safety issues.

### Frequency and plans for auditing trial conduct {23}

The study will be monitored and audited by the study Sponsor, East London NHS Foundation Trust, in accordance with procedures approved by Noclor. A senior trial manager and trial monitor from the PCTU will oversee the monitoring and audit process. A trial monitoring plan will be developed based on a risk assessment, which will specify the frequency of monitoring visits to be conducted by an independent member of the PCTU. The monitoring plan will be agreed by the CI, PCTU and Sponsor.

### Plans for communicating important protocol amendments to relevant parties {25}

Amendments will be submitted to the Sponsor for assessment, categorisation and approval, prior to submission to the Health Research Authority and REC where necessary. The amendment history will be tracked via version and date control of the protocol and associated documents.

## Dissemination plans {31a}

Dissemination activities will run throughout the course of the trial with results disseminated to key stakeholders. The LEAP will take an active role in advising on and assisting with dissemination to ensure findings are accessible and meaningful to patients, carers and the general public. We will target different stakeholders including mental health service commissioners, clinicians, patients, carers and academics.

A project website has been developed and will be regularly updated. The site will contain study information and access to further resources. We will use social media (including twitter and blogs) to communicate research progress, milestones and upcoming events. We will also report progress through NHS Trust newsletters and user publications.

On completion of the trial, the final data will be analysed, tabulated and a final study report prepared for publication in the open access NIHR HTA journal. Further publications will be submitted to peer-reviewed journals and we will present at national and international conferences, including NHS-specific meetings. Lay summaries of findings will be made available via the study website and disseminated to local patient and public groups. Participants will be informed of study results via an end-of-study summary. We will run workshops with arts therapies and NHS professionals at the study end in conjunction with our LEAP.

## Discussion

This will be the first randomised controlled trial to account for patient preferences and diagnostic heterogeneity in group arts therapies. Overall, the study will provide evidence as to whether there is an additive benefit or not to the use of the arts in group therapy in community mental health care. As with many trials of group therapies, there are a number of logistical challenges to which we have had to further adapt, especially due to the COVID-19 pandemic.

Arts therapists are trained in a variety of methods and theoretical backgrounds. Our initial work to develop a shared manual for group arts therapy interventions [28] is now being implemented for the first time within this trial. Drawing upon learning from wider arts therapies and psychotherapy trials [51–54], we have been proactive in offering adherence feedback sessions to arts therapists in the aim of ensuring good treatment fidelity and building therapist confidence. End-of-therapy interviews with therapists [36, 54, 55] will also assist us in learning from the group processes and experiences of implementing the manual within this trial.

We originally designed the study to randomise once a single large cohort of participants was recruited at each site. However, this incurred delays for participants and would have made provision of a large number of therapy groups all at once logistically challenging. To counter this we amended the protocol to enable baseline assessments to happen at the informed consent stage (thus removing an additional burden of assessment) and enabled randomisation immediately once an arts preference quota of 20 was filled. This enabled us to be more responsive and provide therapy more quickly to enrolled participants. However, this has also had limitations in that it has been a challenge to know exactly how much of an arts therapy resource may be required from services as this is based purely on the preference selections made by enrolled participants.

The COVID-19 pandemic hit the study in March 2020 and lockdown measures put in place by the UK government required us to cease all recruitment and group interventions for 16 months. At this time-point, we had a number of participants who we had just randomised but had not yet informed of their group allocation or commenced treatment. During this time frame, study researchers stayed in regular contact with enrolled participants to prevent attrition and monitor for adverse events, surveyed enrolled participants for their views on accessing groups during the pandemic and collaborated with clinical service directors to draw up stringent infection control procedures for face to face groups. We put in place an amendment to allow for remote consent. For those enrolled and randomised at the point of study pause, therapy groups were delayed by more than a year after baseline. We therefore sought an amendment to re-assess eligibility, repeat baseline measures and re-randomise these participants. Recruitment was able to recommence in July 2021 and with the support of clinical services, has successfully resumed.

In spite of the above challenges, the study has continued to recruit successfully to date and is on track to meet its target of 420 enrolled patients.

## Trial status

Protocol version: V8.0, 30 October 2022.

Date recruitment began: 01 February 2019.

Expected date of recruitment completion: 10 February 2023.

## Abbreviations

ADMP: Association for Dance Movement Psychotherapy
AE: Adverse event
AOT: Assertive outreach team
BACP: British Association for Counselling and Psychotherapy
BPRS: Brief Psychiatric Rating Scale
BSI: Brief Symptom Inventory
CI: Chief Investigator
CMHT: Community mental health team
CRF: Case Report Form
CSO: Clinical Studies Officers
CSRI: Client Service Receipt Inventory
DMEC: Data Monitoring and Ethics Committee
EIS: Early intervention services
FGES: Ferrara Group Experiences Scale
GCP: Good Clinical Practice
GSI: Global Severity Index
HCPC: Health and Care Professions Council
HIPC: Humanistic Integrative Psychotherapy College
ICER: Incremental cost-effectiveness ratio
LEAP: Lived Experience Advisory Panel
MANSA: Manchester Short Assessment of Quality of Life
NHS: National Health Service
ORS: Outcome Rating Scale
PI: Principal Investigator
PCTU: Pragmatic Clinical Trials Unit
PHQ-9: Personal Health Questionnaire
PSA: Professional Standards Authority
REC: Research Ethics Committee
SAE: Serious adverse event
SIX: Objective Social Situation Scale
SOP: Standard Operating Procedure
TMG: Trial Management Group
TSC: Trial Steering Committee
UK: United Kingdom
UKCP: United Kingdom Council for Psychotherapy
